# Chocolate and Cocoa-Derived Biomolecules for Brain Cognition during Ageing

**DOI:** 10.3390/antiox11071353

**Published:** 2022-07-12

**Authors:** Corinna Zeli, Mauro Lombardo, Maximilian Andreas Storz, Morena Ottaviani, Gianluca Rizzo

**Affiliations:** 1 Independent Researcher, Via Emo 8, 20128 Milano, Italy; corinna.zeli@icloud.com; 2 Department of Human Sciences and Promotion of the Quality of Life, San Raffaele Roma Open University, 00166 Rome, Italy; mauro.lombardo@uniroma5.it (M.L.); morena.ottaviani@uniroma5.it (M.O.); 3 Center for Complementary Medicine, Department of Internal Medicine II, Faculty of Medicine, University of Freiburg, 79106 Freiburg, Germany; maximilian.storz@uniklinik-freiburg.de; 4 Independent Researcher, Via Venezuela 66, 98121 Messina, Italy

**Keywords:** cocoa, cacao, chocolate, flavanols, cognition, ageing, nutraceuticals, Alzheimer’s disease, MCI

## Abstract

Cognitive decline is a common problem in older individuals, often exacerbated by neurocognitive conditions, such as vascular dementia and Alzheimer’s disease, which heavily affect people’s lives and exert a substantial toll on healthcare systems. Currently, no cure is available, and commonly used treatments are aimed at limiting the progressive loss of cognitive functions. The absence of effective pharmacological treatments for the cognitive decline has led to the search for lifestyle interventions, such as diet and the use of nutraceuticals that can prevent and limit the loss of cognition. Cocoa and chocolate are foods derived from cocoa beans, commonly used in the population and with good acceptability. The purpose of this review was to collect current experimental evidence regarding the neuroprotective effect of chocolate and cocoa (or derived molecules) in the elderly. From a systematic review of the literature, 9 observational studies and 10 interventional studies were selected, suggesting that the biomolecules contained in cocoa may offer promising tools for managing cognitive decline, if provided in adequate dosages and duration of treatment. However, the molecular mechanisms of cocoa action on the central nervous system are not completely understood.

## 1. Introduction

“At no other time has Nature concentrated such a wealth of valuable nourishment into such a small space as in the cocoa bean”

—***(Alexander Von Humboldt)***.

Cocoa (powder) and chocolate can be obtained from Theobroma cocoa beans, which are widely used for human consumption. These products are rich in flavonoids—antioxidant molecules that represent the largest subclass of food polyphenols [1]. Polyphenols are secondary metabolites in plants and have more than one phenol group in their molecular structure.

Flavanols are a subclass of flavonoids found in pome fruits, legumes, green tea, cocoa and wine. In Europe, the intake has been estimated to be 428 ± 49 mg/day, of which 136 ± 14 mg/day is in the monomeric flavan-3-ols form [2]. (−)-Epicatechin is the most abundant monomeric flavanol in cocoa powder. The single units (catechins) combine oligomers and polymers. The longer-chain oligomeric forms are the procyanidins [3]. In addition to flavanols, cocoa contains about 380 known molecules, 10 of which are psychoactive compounds, such as methylxanthines (e.g., caffeine and theobromine) [4].

The antioxidant activity of cocoa beans is even higher than green tea, red wine and blueberries [5], so the human health effects of cocoa products could result from the mix of a high level of antioxidant molecules, despite the strong interest in flavan-3-ols. Flavonoid contents in cocoa products could be influenced by the cocoa cultivars, geographic origin, cultivation and processing [6,7]. Cocoa and chocolate differ not only by polyphenol profile; cocoa is a powder grounded from cocoa beans, while chocolate contains a combination of ingredients that include cocoa, cocoa butter, sugar and other constituents formed into a solid food product [8]. The amount of cocoa in chocolate ranges from approximately 7–15% in milk chocolate to 30–70% in dark chocolate [3]. Furthermore, the bio-availability of cocoa flavanols seems to be influenced by the complexity of the food matrix, so the absorption of these compounds after dark chocolate intake may be less efficient as compared with cocoa powder [9]. Moreover, 100 g of dark chocolate contains about 100 mg of flavanols, while 100 g of unsweetened cocoa powder in non-alkalized form can contain up to 250 mg of flavanols [10]. 

Some human studies showed that the consumption of a specific mixture of flavanols found in cocoa can induce vasodilation of the peripheral and cerebral vascular systems, increasing brain blood flow and perfusion [11,12,13,14]. These data raise the possibility that the regular consumption of cocoa flavanols may have positive implications for cognitive dysfunction. Ageing is usually accompanied by declines in multiple domains of cognitive function, such as memory and processing speed [15]. The average age of the population in Western countries, with an expected increment in low and middle income, is increasing. Thus, the social and individual costs associated with age-related disease are priority problems for both the World Health Organization and the European Union. Cognitive ageing is becoming a major public health concern worldwide [16,17]. 

Dementia and its main form, represented by Alzheimer’s disease (AD), are prominent causes of accelerated cognitive decline (CD) [17]. Currently, there are no safe and affordable approved interventions to prevent CD in old adults. Insulin and insulin receptors are involved in brain signaling, neural growth and survival [18]. Taking into account that patients with diabetes have a higher risk of AD incidence, diet seems a good candidate as a modifiable factor for prevention and treatment [19]. Some studies suggest that reduction in dietary fats is a potent measure to reduce the risk from AD [20], so the chance of early prevention (or slowing) through diet management and nutritional practices may be of critical importance [21]. In recent years, research in this area has increasingly focused on the development of nutraceuticals and lifestyle approaches. Oxidative stress and inflammation are the hallmarks of dementia and play a role in the cognitive decline with age, called “inflammageing”, including multiple mechanisms, such as epigenetic alterations and genomic instability, affecting intracellular functions and intercellular communication [22]. These dysfunctions could be mediated by dysregulated deposition of neurotoxic molecules, such as beta-amyloid or hyperphosphorylated tau peptides in neurodegenerative diseases [23,24]. Cocoa flavonoid consumption may lead to anti-inflammatory and antioxidant effects to counteract neuronal degeneration and neuroinflammation affecting cognitive functions [25,26]. Chocolate consumption is generally considered a pleasant widespread habit, qualifying it as a good candidate for daily phytochemicals supplement, despite the drawbacks related to the high caloric intake associated with it. This idea was supported by several epidemiological studies emphasizing potential benefits of flavonoid-rich foods on CD and the risk of dementia [27,28,29]. Moreover, growing evidence suggests an influence of microbial-derived metabolites on neurological function, given the low bioavailability of parent polyphenols, highlighting a role of gut microbiota in cognition [30,31].

Human trials have revealed that tests of executive function and processing are sensitive to cocoa flavanols [32]. Moreover, chocolate, cocoa supplements or cocoa flavonoids in acute or sub-acute administration showed cognitive improvement [13,33,34,35]. The potential effects of cocoa or chocolate in improving or preventing CD remain unclear, so there is no consensus on a nutrition-based preventive strategy.

The scope of this review is to systematically and comprehensively collect available observational and interventional data about the role of chocolate, cocoa and derived phytochemicals in cognition among the elderly.

## 2. Methods

A systematic consultation of literature was launched on 4 search engines (PubMed (https://www.ncbi.nlm.nih.gov/) (accessed on 14 March 2022), ScienceDirect (https://www.sciencedirect.com/) (accessed on 14 March 2022), Cochrane Trials Library (https://www.cochranelibrary.com/) (accessed on 14 March 2022), ClinicalTrials.gov (https://clinicaltrials.gov/) (accessed on 14 March 2022)) using the following query: (theobroma OR chocolate OR cocoa) AND (cognition OR cognitive OR dementia OR Alzheimer).

We searched the keywords in titles, abstracts and authors’ keywords and combined them with available MeSH terms. The query was adapted to the search engine used. No filters from the search engines were used, and results were collected from the inception through to 14 March 2022. The obtained entries were screened for duplicates and then screened by titles and abstracts. After that, the full texts were retrieved for evaluation and inclusion in the summary.

Editorials, letters to the editor, commentary, reviews, meta-analyses, case series, case studies, non-human studies, in vitro studies, conference papers, methods, chapters, non-English papers, studies that only included people under the age of 65 and studies with outcomes not pertinent to cognition were excluded. The procedure was carried out following the most recent PRISMA guidelines [36]. 

## 3. Results and Discussion

**Figure 1:**
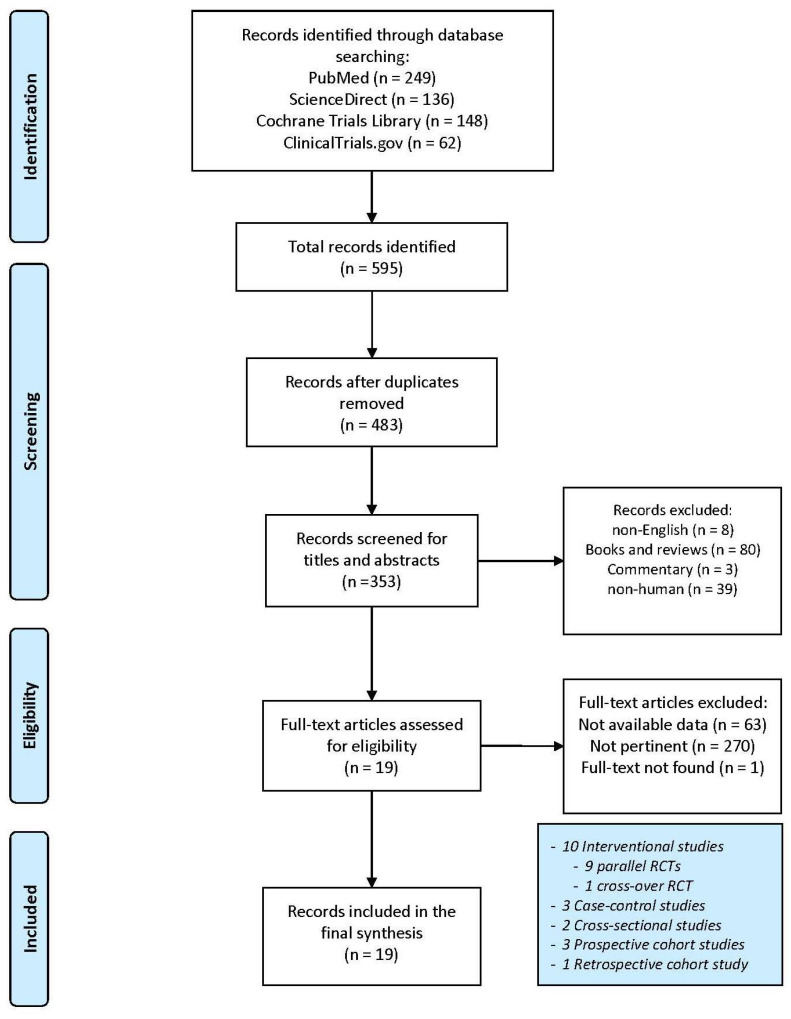
Flow chart for study selection. Adapted from Moher et al. [37].

A total of 595 articles were obtained from the search engines (PubMed: 249; ScienceDirect: 136; Cochrane Library Trials: 148; ClinicalTrials.gov: 62). After the removal of 112 duplicates, the selection was carried out through titles, abstracts and full-text reading. A total of 19 entries were identified and used for the final synthesis. The detailed selection process is highlighted in Figure 1.

The final selection included one retrospective study, two cross-sectional studies, two prospective cohort studies, three case–control studies, nine parallel, randomized controlled trials (RCTs) and one cross-over, randomized controlled trial. 

### 3.1. Observational Studies

All selected observational studies investigated the correlation between chocolate consumption and cognitive functions in old people, showing a rather protective role of chocolate intake on CD. The dietary habits were assessed by self-administered food frequency questionnaires related to the previous year. Cognitive performance was usually measured with a test battery and mini-mental state examination (MMSE). One common limitation among the studies could be that participants with impaired cognition may have altered their diet as a consequence of cognitive status, so their self-reported dietary data may be less reliable.

Two cross-sectional studies were performed in 2009 by Nurk et al. and in 2016 by Crichton et al. [38,39]. Cognitive functions were evaluated with a battery of neuropsychological tests based on global cognition, visual-spatial memory, semantic or episodic memory and working memory. 

Nurk et al. examined the intake of three common foods containing flavonoids (chocolate, wine and tea) in a population of 2031 participants between 70 and 74 years old within the Hordaland Health Study in Norway [38]. The population’s mean intake of chocolate was less than 8 g/d, with a maximum beneficial effect on cognitive performance at an intake of 10 g/d. The associations between the intake of these three foods and cognition were synergic and dose dependent because there was no effect of each food or beverage analyzed separately.

On the other hand, Crichton et al. investigated whether chocolate intake was associated with cognitive function in a smaller community-dwelling cohort of 968 participants from the Maine-Syracuse Longitudinal Study (MSLS) [39]. The cohort included participants aged 23 to 98 years. Serving sizes were not reported by participants to define the proper chocolate consumption, but all cognitive test scores were significantly higher in those who consumed chocolate at least once per week compared with those who never/rarely consumed chocolate. The relationship was not attenuated when adjusted for cardiovascular, lifestyle and other dietary factors.

These results suggested that habitual chocolate intake was related to better cognitive performance. It is important to take into account that cognition in humans is shaped by long-term exposures, and this can limit the strength of data from cross-sectional studies.

In 2016, Moreira et al. selected a cohort of 531 participants living in Porto, aged > 65 years, with normal MMSE basal scores, to perform a longitudinal prospective study [40]. After a median follow-up of 48 months, the CD was defined by a decrease of at least 2 points in the MMSE score. Chocolate intake was associated with a lower risk of CD (RR = 0.59, 95% CI: 0.38–0.92). After stratification, a lower risk of CD was statistically significant for levels of chocolate corresponding to an average weekly intake lower than one standard portion (defined as three pieces of a chocolate bar, one chocolate snack or one tablespoon of cocoa powder). 

As we described in Nurk and coworkers’ study described above, in Moreira and colleagues’ study, the protective effect of chocolate also appears synergic with the consumption of another food. Indeed, the positive effect was observed only among subjects with an average daily consumption of caffeine lower than 75 mg. 

A landmark study in the field, this was the first prospective cohort study on old cognitively intact people that showed an inverse association between regular long-term chocolate consumption and CD.

In a 2020 case–control study, Filippini et al. reported an inverse association between early onset dementia (EOD) risk and chocolate product consumption [41]. They compared dietary habits using a food frequency questionnaire and adherence to some dietary patterns of 54 newly diagnosed EOD patients in an Italian population compared with 54 caregivers as controls, with a mean age of about 65 years old. Despite photos of serving sizes being used to assist the choice of participants, the portions are not to be considered certain. Chocolate-based products showed a lower EOD risk associated with moderate and high intake (>20 g) of chocolate per day. However, some limitations can be highlighted: the small samples of participants may have affected the power of detection; the use of caregivers as controls could have led to a risk of overmatching and minimized the real associations between dietary factors and disease risk. Moreover, some controls (particularly cases’ family members) may have shared lifestyle habits, including dietary patterns, with EOD cases. 

In 2021, Zhong et al. conducted a post hoc analysis of the prostate, lung, colorectal and ovarian (PLCO) cancer screening trial to examine the association between chocolate consumption and mortality in a US population [42]. The analysis comprised a large population-based cohort of 91,891 participants, aged 55 to 74 years, and an average follow-up of 13.5 years. They found that chocolate consumption was inversely associated with the risk of death from all causes and cardiovascular disease in a nonlinear dose–response manner. Indeed, the lowest risk observed was at chocolate consumption of 0.7 servings/week and 0.6 servings/week, respectively, with 1 serving size defined as 28.35 g of chocolate. In a fully adjusted model, chocolate consumption was found to also be inversely associated with the risk of mortality from AD (HR for >2 vs. 0 servings/week 0.69; 95% CI: 0.49–0.99; *p* = 0.041). No significant association was found for cerebrovascular disease. Chocolate consumption was assessed by a self-administrated food frequency questionnaire at baseline in this study. Yet, the data did not allow for a differentiation of milk and dark chocolate. The assessment of dietary exposure at a single time point may be controversial, given that dietary habits are likely subject to change over time. These findings were derived from a US population with a relatively high chocolate consumption (the average chocolate consumption was 1.5 ± 3.2 servings/week) and thus might not be extended to other populations with lower chocolate intakes.

Very few studies have investigated cognitive ageing from a global long-term perspective, which may be an interesting approach to capture predictors between early dementia stages and normal cognitive ageing. To better understand which nutritional biomolecules are related to a lower risk of dementia and CD, some epidemiological studies investigated metabolic changes using a metabolomics approach. Metabolomics provides a global picture of an individuals’ biological status, and it provides the identification of new pathways in the human metabolome, directly derived from food digestion. 

In 2019, Low et al. applied untargeted metabolomics analyses on the serum of participants from a large French population-based cohort (Three-City study), with no cases of dementia at the time of blood draw (baseline), and provided repeated measures of cognition over 12 years [43]. A nested case–control study was performed on volunteers aged ≥ 65 years. A total of 209 cases with greater CD were matched with 209 controls with slower CD (based on age at baseline, gender and educational level). A total of 22 serum metabolites at baseline were associated with subsequent CD. The signature included the cocoa metabolite cyclo(prolyl-valyl) (C10H16N2O2), derived from roasted cocoa beans. Interestingly, theobromine was correlated with chocolate intake (r = 0.20) and, although not selected in the 22 metabolite signatures, was negatively associated with CD (univariate OR for 1SD increase: 0.85). These data suggest that chocolate consumption could be associated with slower CD.

From the same prospective cohort study, in 2021, González-Domínguez et al. performed two separate nested case–control sample sets from different geographic regions (Bordeaux, n = 418; Dijon, n = 424) [44]. Cases were chosen between the participants with the worst score of CD (209 cases in Bordeaux and 212 cases in Dijon). Each case was matched to a control (i.e., a participant with a score of CD better than the population median) with the same age, gender and education level. The food-related and microbiota-derived circulating metabolome was studied using serum samples for large-scale quantitative metabolomics analysis. In line with the previous untargeted metabolomics study of Low and colleagues, they observed an inverse association between 3-methylxanthine (a metabolite derived from cocoa theobromine) and subsequent CD. 

Around 90% of proanthocyanidins are non-absorbable, and the colon is the key organ for their biotransformation [45]. Indeed, various phenolic microbial metabolites were detected in human urine after the consumption of soluble cocoa powder [46]. The production of these microbial-derived compounds with a high rate of absorption is unpredictable because it depends on the intestinal microflora of different volunteers undergoing different enzyme metabolization. So, it seems plausible that adequate gut competence associated with microbial action could contribute to bioavailability of cocoa biomolecules, improving their systemic action. In the study of Low and colleagues [43], conjugated secondary bile acids were associated with CD. This phenomenon further suggests the possible link between dysfunction in brain lipid metabolism, increased blood concentration of specific subpopulation of bile acids and AD [47,48]. Given the interplay between gut microbiota and bile acids, gut microbial ecology could act in a way, which favors or limits bioavailability of brain-active molecules.

All these observations reinforce the beneficial effect of cocoa consumption against CD.

Among the selected observational studies, only one failed to find a correlation between cognitive functions and chocolate consumption. In 2018, Haller et al. explored the correlation between regular consumption of chocolate, wine and coffee with the structure and cerebral blood flow in the elderly [49]. In total, 145 community-based individuals aged from 69 to 86 years were included and underwent neuropsychological assessment and magnetic resonance imaging (MRI) during a subsequent 3-year period. All individuals were evaluated with an extensive neuropsychological battery, including the MMSE. Participants without dementia or MCI were classified as cognitively healthy controls and underwent full neuropsychological assessment at follow-up, on average 18 and 36 months later. After the assessment, individuals were classified into three groups: stable, intermediate or deteriorating. The usual food and beverage consumption was assessed by a self-administered food questionnaire. Participants were asked to complete the questionnaire by entering the amount of consumed foods per day, month and year. Monthly consumption of chocolate was divided into light, moderate and heavy, but the analysis did not show any correlation. Chocolate consumption, in any frequency, was not associated with cognition or MRI.

We also included in the final synthesis one longitudinal study regarding cocoa flavanols supplementation [50]. In vivo activity of polyphenols could be reduced by the chemical structure, the isomerism, polymerization and the interaction with other compounds [51]. However, a high concentration of cocoa-derived flavonoids from supplements and nutraceuticals could act in synergy with other compounds and have a potential pharmacological effect on the slowdown of neurodegenerative processes.

In 2019, Calabrò et al. performed a retrospective cohort study including 55 Italian patients (29 males and 26 females, aged 56–75) with a new diagnosis of amnestic mild cognitive impairment (MCI), a subtype of MCI that is more likely to convert into AD [50]. The study aimed to evaluate the effect of cocoa polyphenols on cognition after a one-year follow-up. Dietary supplementation of 240 mg of cocoa flavonoids reduced the progression of MCI to dementia, evaluated with the MMSE score. In particular, the Mexenion^®^ intake, a polyphenols-rich cocoa nutraceutical commonly used to treat MCI, was significantly higher in patients without a worsening in cognition after one year.

Table 1 summarizes the study characteristics of the selected observational studies.

### 3.2. Interventional Studies

In its natural form, non-alkalized cocoa powder contains high amounts of a subtype of flavonoids called flavanols, mainly epicatechin, catechin and their oligomers. It also seems that flavanols monomers are the most bioavailable compared with other dietary flavonoids [52].

In most of the following trials, cocoa powder was enriched with additional flavanols, and it was consumed as a drink [53,54,55,56,57,58,59,60]. In some trials, cocoa powder was mixed in a dairy-based [53,55,56,57] or non-water matrix [59]. All cocoa drinks were usually standardized for their total cocoa flavanols content and closely matched for macro- and micro-nutrient content and caloric level. Intervention drinks were provided by corporates as dry cocoa beverage mixes. Additionally, all drinks were similar in taste and appearance and were supplied in individual sachets labeled with an anonymous code. Volunteers were asked to consume drinks immediately after preparation. 

Participants were usually, but not always, advised to not alter their usual energy intake, and a nutritionist instructed them to keep their energy intake in balance through substitution of their sweet snacks with the intervention drink. Participants, usually but not always, received guidance to limit the intake of polyphenol-rich food items, such as wild berries and apples, tea, red wine, fruit and vegetable juices and chocolate other than the intervention doses. Finally, they were asked to document any unusual symptoms or side effects and to keep a diary of illness and medications. Notably, the study design described above was not employed in all studies, potentially contributing to heterogeneity in the results.

Impairment of endothelial function and decreased cerebral perfusion are elements of the ageing process and correlate with dementia. Peripheral vascular disease and cerebrovascular disease are responsible for significant mortality with advancing age [61]. Cocoa flavanols seem to affect endothelial vascular function [62,63]. We included two short-term interventions with flavanol-rich cocoa in old volunteers that suggested improvements in cerebral blood flow (CBF) and neurovascular coupling [54,57].

In 2008, Sorond et al. conducted an interventional study on the effects of cocoa flavanols consumption on CBF in old healthy volunteers. They administrated a flavanol-rich cocoa (FRC) drink, with 900 mg flavanols, to thirteen subjects (72.5 ± 4 years old) for two weeks [57]. At first, they investigated the effect of acute and short-term regular cocoa intake in a single-arm intervention. Transcranial Doppler ultrasound (TCD), in response to one week of regular FRC intake, showed an increase in the mean cerebral blood flow velocity of 8% ± 4% (*p* = 0.01). After two weeks, the increase was 10% ± 4% (*p* = 0.04). 

Afterward, twenty-one subjects (72.2 ± 6 years old) were randomized in a double-blinded, parallel-arm placebo-controlled study designed to compare the effect of one week of dietary intake of FRC vs. flavanol-poor cocoa (36 mg flavanols per day) on mean cerebral blood flow velocity. Although the FRC group had a larger per cent increase in their mean CBF (54% ± 3% on FRC vs. 16% ± 2% on FPC), these intergroup differences were not statistically significant due to the larger variability in the responses.

The link between neurovascular coupling (NVC) and cognition or structural brain changes that occur with ageing remains unknown [64]. NVC expresses the relationship between the increase in CBF and the increase in oxygen and glucose delivery to promote a better neuronal activity. 

In 2013, Sorond et al. investigated the relationship between NVC and cognitive function in old individuals with vascular risk factors to determine whether NVC could be modified by cocoa consumption [54]. Cerebral blood flow velocity (BFV) was measured before and after 30 days of a flavanol-rich cocoa (609 mg, two times a day) vs. a flavanol-poor cocoa (13 mg, two times a day) drink. Blood flow and blood pressure changes were not significantly different between the two cocoa groups. Similarly, there was no difference in change in NVC. Yet, improvements were present in both groups. Cognitive assessment was performed with MMSE and trail making tests A and B (as a measure of executive function). After 30 days of cocoa consumption, the only association observed was the increased neurovascular coupling (5.6% ± 7.2% vs. −2.4% ± 4.8%; *p* = 0.001) and improved trails B times (116 ± 78 s vs. 167 ± 110 s; *p* = 0.007) in those with impaired neurovascular coupling at baseline. This double-blind clinical trial showed that cocoa consumption resulted in higher NVC and that individuals with higher NVC had a better cognitive function. The findings explained above are limited to an old cohort with vascular disease and should not be interpreted as applicable to the physiology of ageing in a healthy population. No significant effects of cocoa administration on neurovascular coupling and cognitive performance were observed in those with intact cerebrovascular functions. The dose–response curve and the kinetics of cocoa flavanols acting on cerebral blood flow are still unclear. Future studies in this field should be dedicated to untangling these various mechanisms. 

Some studies showed that the regular intake of cocoa flavanols can improve aspects of cognitive performance among old subjects, and this phenomenon appeared to be dependent on the amount of cocoa flavanols intake (intermediate or high flavanols).

In 2012, Desideri et al., in a parallel double-blind trial, tested the hypothesis that dietary flavanols might improve cognitive function in subjects with mild cognitive impairment (MCI) [53]. The neuropsychological function was assessed in 90 old persons with a mean age of 71 years old, randomized to consume a high-flavanol (990 mg/day) or an intermediate (520 mg/day) drink, for 8 weeks. At the end of the follow-up period, in the high- and intermediate flavanol compared to the low-flavanol groups, the times required to complete both trail making tests (TMT) A and B were significantly lower. The verbal fluency score was significantly better in the group with a flavanol intake. The high- and intermediate flavanol groups also exhibited decreased insulin resistance, blood pressure and lipid peroxidation. The study found that cognitive performance was improved with regular cocoa flavanol consumption, without evidence of any relevant adverse effects, and no significant differences in body weight or body mass index were observed. These improvements were already evident at an intermediate cocoa flavanol content, not only for high intake, suggesting a particular sensitivity of this subset of neuropsychological functions to the benefits of cocoa flavanols. On the other hand, the MMSE did not show an intervention-related effect, perhaps reflecting the low sensitivity of this test to detect small changes at the upper end of cognitive performance over time. Improvements in some metabolic markers, such as the decrease in insulin resistance, suggest a possible role of glucose metabolism in modulating cognitive function in these subjects. It is yet unclear whether the observed benefits in neurocognition are a direct consequence of cocoa flavanol or secondary to a general improvement in cardiovascular function.

Mastroiacovo et al. (2015) randomly assigned 90 healthy subjects aged 61–85 into three groups that received a daily drink containing low (48 mg), intermediate (520 mg) or high (993 mg) amounts of cocoa flavanols [55]. The remaining bioactive composition of the drink, including caffeine and theobromine, was matched across the three interventions. After 8 weeks, both the intermediate and high-flavanol groups showed improvements in processing speed and attentional tasks (measured by the TMT); improvements in verbal fluency (VFT) were greatest for the high-flavanol group. MMSE was again not significantly different between groups post-treatment. Participants also improved with cardiometabolic variables. 

In these two RCTs, the daily consumption of flavanol-rich cocoa drink was shown to positively affect cognition, leading to improvements in cognitive performance both in old adults with early memory decline and in apparent cognitively intact subjects.

The study by Mastroiacovo and colleagues also suggests that high-cocoa-flavanols-containing foods could potentially be effective in reversing certain aspects of age-related CD. However, the observed cognitive improvement could be a consequence of alkaloids (50 mg caffeine and 400 mg theobromine). These observations were obtained in a healthy population but were similar to the results described by Desideri et al. in individuals with MCI [53].

These findings suggest a potentially restorative but not preventive effect of habitual flavanol consumption on different cognitive dysfunctions in old subjects.

Crews (2008) and Suominen (2020) were the only randomized controlled trials investigating the effects of dark chocolate naturally high in cocoa flavanols on cognitive functions [58,65]. Crews et al. (2008) studied the effects of 37 g dark chocolate bars (397 mg flavanols) plus a cocoa beverage (357 mg flavanols) on neuropsychological functions and cardiovascular health in a group of 90 healthy subjects over 60 years of age in a six-week intervention [58]. The study failed to support the predicted beneficial effects on any of the neuropsychological, hematological or physiological variables included in the investigation. The authors hypothesized that cognitive changes were not manifest in healthy participants without cognitive impairment. However, the sample size could have decreased the power of detection, along with the short duration of the treatment.

Suominen et al. (2020) studied the effects of commercial 50 g dark chocolate pralines (410 mg flavanols) on neuropsychological functions, blood lipids and glucose in healthy subjects with a mean age of 69 years old in an eight-week intervention [65]. Cognition was assessed with the verbal fluency test (VFT) and TMT A and B. No differences in cognition were seen between the groups. Their intervention improved neither cognitive functions nor cardiovascular health after short-term dark chocolate consumption. 

The type and bioavailability of cocoa flavanols may differ in enriched products, such as drinks, compared to dark chocolate naturally high in cocoa flavanols, which may have affected some study results. Additionally, the participants of both of these studies had a healthy cognitive status and metabolic profile and a high education level. Moreover, at baseline, they performed cognitive tests with excellent results. The recruitment process was very easy due to the high interest in an intervention concerning chocolate, so the participants might have been a selected group of people that are especially interested in their health and nutrition. In Crews and Suominen and colleagues’ studies, an insufficient flavanols content in a chocolate bar or a different bioavailability may, therefore, explain the results.

The most used neuropsychological tests to measure outcomes in selected studies were the TMT A and B and the VFT. The combination of these is feasible for this type of study and the most sensitive for measuring changes in intervention among older adults. The same cognitive measurements have been used by Desideri et al., 2012, Sorond et al., 2013, Mastroiacovo et al., 2015 and Suominen et al., 2020 [53,54,55,65]. 

Neuropsychological tests can evaluate the indirect association between cocoa flavanol intake and cognitive performance but are insufficient for providing information about the direct mechanisms of action of cocoa flavanols. The potential mechanisms can involve improvements in the cerebral blood volume, as described above, and/or an increase in serum neurotrophin concentration, such as brain-derived neurotrophic factor (BDNF).

In 2016, Neshatdoust et al. investigated the link between changes in BDNF and human cognitive performance in old subjects following high cocoa flavanol intake [56]. All 40 volunteers were assessed as “healthy” with a mean age of 68.3 years. The study was a cross-over dietary intervention, in the course of which subjects were asked to take either a high-flavanol cocoa drink (494 mg total flavanols daily) or a low-flavanol cocoa drink (23 mg total flavanols) for 28 days. After the first intervention period, there was a 4-week wash-out period before switching volunteers to the alternate intervention of the study. A significant increase in global cognition due to a high cocoa flavanols intake (*p* < 0.01) was observed along with changes in serum BDNF levels (*p* < 0.01), suggesting a role for BDNF in flavonoid-induced cognitive improvements.

On the contrary, no change in neurotrophin levels was found in 2021 by García-Cordero et al. [59]. They analyzed the effect of cocoa flavanols and red berry anthocyanins on BDNF and nerve growth factor receptor (NGF-R), and they tried to determine the possible improvement in cognitive performance by using a battery of neuropsychological tests. The study was performed on 60 healthy volunteers aged 50 to 75 years who consumed cocoa powder (200 mg of flavanols per day), a red berries mixture or a combination of both for 12 weeks. The flavanols’ dietary dose was significantly lower compared to that of cocoa drink used by Neshatdoust and coworkers’ intervention [56]. However, an improvement in executive function (enhancing performance on the neurocognitive test Tower Of London) was shown in all groups, in particular for the intervention with the combination of cocoa and red berries. The authors found a correlation between high BDNF levels and better task performance (fewer movements required to finish the TOL) in women. Additionally, they found a positive correlation between higher levels of NGF-R and the time required to finish a working memory test in both sexes.

In 2014, Brickman et al. conducted a flavanol-rich cocoa intervention to enhance dentate gyrus (DG) function, which, in turn, would improve cognition in the hippocampal circuit during ageing [60]. In a controlled randomized trial, they studied 41 healthy 50–69-year-old subjects (37 participants completed the study) who consumed either a high- (900 mg flavanol daily) or low- (45 mg flavanol daily) cocoa-containing diet for 3 months. As measured with functional MRI (fMRI), the high-flavanol intervention enhanced DG function, increasing cerebral blood volume in the related hippocampus region. These findings therefore suggest that DG dysfunction could be a driver of age-related CD.

In 2021, Sloan et al. tried to replicate and extend the results from Brickman’s previous study into a larger sample, propose a range of flavanol intake amounts and assess the persistence of effects on cognitive function related to multiple brain regions [66]. They conducted a controlled, randomized, parallel-arm dietary intervention with 211 healthy adults (50–75 years old), investigating the effects of either a placebo or 260, 510 and 770 mg/day of cocoa flavanols for 12 weeks followed by an 8-week washout. The object-recognition task localized to the hippocampus’ dentate gyrus did not improve after flavanol intake, as opposed to what Brickman showed in 2014 in a smaller group. Hippocampal-dependent list-learning performance task was directly associated with baseline diet quality and improved after flavanol intake. This observation was particularly significant in participants in the lower tertile of diet quality. In this cohort of healthy individuals, those with lower alternative healthy eating index (aHEI) scores at baseline experienced a greater treatment effect. These findings suggest that dietary flavanols may be associated with memory functions of the ageing hippocampus and normal CD. 

The selected clinical trials of this review did not show any weight difference among participants after the consumption of cocoa and derivatives, where investigated [53,54,55,59,65]. However, the interventions were organized to be isocaloric between groups. In the selected observational studies, only two studies evaluated differences in weight or adiposity based on the consumption of chocolate or cocoa [39,42], without showing any clinically relevant differences. In a recent scoping review, the relationship between anthropometric outcomes and cocoa consumption was investigated, showing conflicting results [67]. Some studies showed an improvement in weight and adiposity, while others showed a direct association between weight or adiposity and cocoa or chocolate. However, the selected studies involved only obese adults, thus limiting their generalizability. In a systematic review and meta-analysis on the relationship between flavanols and body composition, the sub analysis for cocoa flavanols showed ameliorative effects on metabolic but non-significant effects on weight and waist circumference [68]. The influence of cocoa and chocolate can act on many metabolic aspects that require further investigation.

A list of the selected interventional studies with their characteristics is summarized in Table 2.

### 3.3. Study Quality 

The work of Sorond et al. in 2008 included two interventions: one longitudinal single-arm intervention and one parallel RCT [57]. At the risk of bias judgment, we only included the latter, given that the former was not randomized. After risk assessment, four trials showed overall high risk, four with low risk and two with some concerns. The randomization process highlighted some concerns in 3 out of 10 studies. However, all studies exerted a low risk of possible bias in reporting results. The work of Crews and colleagues showed a higher risk of bias [58], as emerged from high risk in two domains: deviation from intended intervention and missing outcome data. 

**Figure 2:**
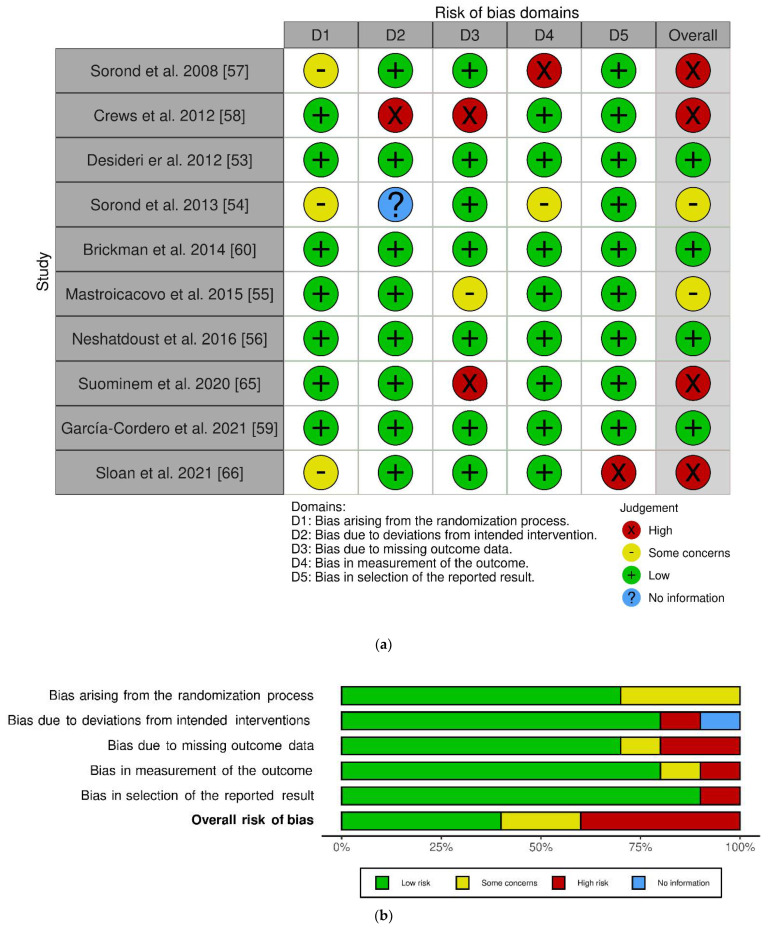
Risk of bias using Cochrane Risk of Bias tool for randomized clinical trials (RoB2) [69]: Individual studies assessment, (**a**); overall assessment, (**b**).

A graphical representation of the risk of bias assessment using the RoB 2 tool can be found in Figure 2 [69]. 

## 4. Proposed Mechanisms of Action

Monoisomers of epicatechin and catechin are the predominant flavonoid compounds in cocoa. They act as a structural element for oligomers—the proanthocyanidins. The antioxidant properties of flavanols are chemically mediated through the oxidation of two aromatic hydroxyl groups to a quinone [70].

Even though flavanols have in vitro antioxidative activity, their brain activity is closely correlated with the accessibility of molecules to the brain. It is known that dietary flavanols do not necessarily have the best bioavailability profile due to environmental, food-related and host-related factors [71]. Bioactive compounds of cocoa flavan-3-ols could improve cognitive function in humans through multiple mechanisms of action.

### 4.1. Neurotrophins 

Catechin and, in a more efficient manner, epicatechin, have been shown to cross the blood–brain barrier (BBB) in human cell lines [72]. The ability of cocoa flavanols to penetrate and accumulate in the brain tissues suggests they could play a direct role in the central nervous system [73]. The above-discussed study by Neshatdoust and colleagues showed the link between cocoa flavanols and the role of BDNF in increased cognition in humans [56].

### 4.2. Cerebrovascular Action 

Cognitive benefits in humans may arise from indirect mechanisms of vasodilatation, improving peripheral and central blood flow, and vascular endothelium protection mediated by nitric oxide. 

Early in vitro data showed the nitric oxide synthase (NOS) activation in vascular endothelium by flavanols [74]. Cocoa flavanols, in particular epicatechin, were shown to increase the bioavailability of the main regulator of vascular function: nitric oxide (NO). This function leads to improvements in vascular tone and blood pressure regulation [75].

In humans, Fisher et al. (2003) performed a study on healthy subjects receiving a flavanol-rich cocoa drink (821 mg of flavanols) daily for 4 days. Peripheral arterial tonometry showed an amplitude increase of about 29%, twelve hours after the last drink. On the 5th day, an additional dose of cocoa led to a 33% increase after 90 min [63]. The mechanism leading to vasodilatation was supposed to be a nitric oxide (NO)-dependent action because a NOS inhibitor administered after 4 days of cocoa consumption completely reversed the amplitude of vasodilatation [63,76].

The rise of NO concentrations in plasma and the improvement of flow-mediated vasodilation were confirmed in human studies assessing the impact of cocoa beverages [77]. Repeated administration of cocoa was demonstrated to bring long-term effects. These cocoa flavanols’ functions act to enhance the baseline level of flow-mediated dilation modulating gene expression and protein synthesis of endothelial nitric oxide synthase (eNOS) [78].

The action of cocoa flavanols on the endothelium can also be linked to the decrease in xanthine oxidase and myeloperoxidase activities, modulation of PGI2 and leukotrienes, inhibition of proinflammatory cytokines IL-1β, IL-2 and IL-8 production, as well as ET-1 release [79].

Cocoa flavanols may be useful in counteracting the decrease in endothelial function associated with ageing not only peripherally but also in the central nervous system [54,57]. Adequate cerebral blood flow (CBF) is important for oxygenation, waste metabolite excretion and glucose distribution to neurons. Greater availability of metabolic substrates can increase neurocognitive functions [61].

The literature also showed that natural products increasing CBF and brain metabolism are effective in enhancing cognitive performance [80]. On the contrary, several studies indicated that there is a decrease in CBF in patients with dementia [19,81].

In humans, some evidence highlights the potential of flavanol-rich cocoa influencing cognitive abilities, interacting with the cerebrovascular system and increasing CBF. As described above, one study found that the ingestion of a single dose or a 1-week treatment with cocoa rich in flavanol (900 mg daily) increased CBF in gray matter and reversed endothelial dysfunction in a dose-dependent manner [82]. Moreover, another study showed an increase in CBF 2 h post-consumption of a single acute dose of 494 mg cocoa flavanols in adults aged 50–65 years. The study employed arterial spin labeling functional magnetic resonance imaging [83].

These acute cognitive enhancements following a single administration in young or middle-aged subjects add up to a long-term cognitive effect of cocoa on old persons discussed in this review. Chronic flavanols intake seems to result in a more efficient cerebrovascular coupling during neuronal activation and improve CBF velocity [54,57].

### 4.3. Insulin Resistance 

Dietary flavanols consumption has been shown to improve insulin sensitivity. In particular, interventions with chocolate or cocoa seem to reduce insulin resistance through a decrease in its pancreatic secretion [84]. However, the exact mechanism is still unclear. This action of cocoa flavanols could be particularly important because insulin sensitivity is a modifiable factor and may have a huge effect on cognitive functions in light of the role of this hormone in modulating brain structure and function [85]. Growing evidence suggests a central role of insulin resistance in certain aspects of brain ageing [86,87]. 

Insulin acts as a vasoactive hormone that modulates both cerebral and peripheral blood flow [88], binding to receptors on endothelial cells, increasing the production of nitric oxide that acts on blood vessels’ tone. However, insulin can alternatively constrict blood vessels at high concentrations [89]. Hyperinsulinemia promotes higher blood pressure and reduced cerebral perfusion; these detrimental effects could be observed in the time before the onset of cognitive symptoms distinctive for vascular cognitive impairment, the second leading cause of dementia after AD [90].

Cocoa flavanols affect both endothelial vascular function and insulin sensitivity through reciprocal mechanisms, which involve nitric oxide [84]. These mechanisms are further highlighted by in vitro studies showing the effects of flavan-3-ols and their metabolites on glucose transport, inflammation and platelet function [77,78,91,92,93].

In the work of Desideri et al. discussed above, the cognitive improvements were demonstrated in parallel with significant improvements in blood pressure and insulin sensitivity [53]. Insulin resistance suggests a possible influential role of glucose metabolism in modulating cognitive function in old persons with mild cognitive impairment. Mastroiacovo also found improvements in insulin resistance, blood pressure and lipid profile, both in the high and intermediate flavanol intake groups, compared to the control, in old cognitively intact subjects [55].

These findings add up to growing evidence suggesting a central role of insulin resistance in certain aspects of brain ageing [86,87]. Furthermore, these data suggest that dietary interventions improving glucose homeostasis may confer important cognitive benefits. 

Changes in the insulin signaling pathway could guide detrimental cognitive processes [86,94,95]. Insulin receptors were found in the BBB and were downregulated from chronic peripheral hyperinsulinemia [96]. This phenomenon causes a low insulin concentration in the brain. This was shown in patients with AD [97].

It was shown that brain insulin resistance together with insulin deficiency occur in MCI patients and early stage AD patients. From these observations, it was proposed that type 3 diabetes could be considered a pure form of brain insulin resistance and a significant causal factor for AD pathogenesis [98]. Thus, insulin resistance in type 3 diabetes may be prodromal of AD. In supporting this type 3 diabetes hypothesis, it has been shown that insulin administration can improve cognitive function in AD patients [99,100].

Results from prospective observational studies about the intake of antioxidants and vitamins in AD patients are conflicting. The hypothesis would be that the beneficial properties of flavanols on cerebral function could allow the delaying of the development of MCI to AD [101]. 

More research in this area is needed to explore this hypothesis further.

### 4.4. Gut Microbiota 

Given the low bioavailability of polyphenols, growing evidence suggests that microbial-derived compounds could be involved, at least in part, in their biological effects.

Catechins are a monomeric form of flavan-3-ols from which polymers, such as proanthocyanidins, are derived. Dietary sources of proanthocyanidins include baking chocolate (1635.9 ± 334.6 mg/100 g) [1]. Molecular size plays an important role in oral bioavailability, and proanthocyanidins have a complex chemical structure. Their bioavailability is very low, and it is poorly understood how they can exert their biological effects. Proanthocyanidins with a degree of polymerization higher than four monomers cannot penetrate the intestinal barrier [45,102]. On the contrary, a high plasma level of monomers (257 nmol/L) was reported in humans after ingestion of 80 g of chocolate, providing 577 mg of proanthocyanidins [103]. 

When discussing the potential interactions between microbial-derived compounds and CD, protocatechuic acid (PCA) is another interesting example. PCA is the main metabolite of several complex polyphenolic compounds (mainly anthocyanins). It is metabolized by intestinal microflora and can be detected both in blood and urine, easily crossing the BBB [104,105,106]. Experimental studies suggest a striking role of PCA in the prevention of neurodegenerative development. Its influence on the processes underlying cognitive and behavioral impairment may promote protective mechanisms against disorders such as Alzheimer’s and Parkinson’s diseases [24].

In light of this, microbial-derived cocoa compounds could be involved in the biological effects attributed to polyphenols on cognitive functions. In the above-discussed work of González-Domínguez et al., the serum levels of metabolite 3-methylxanthine were highly correlated with theobromine (r > 0.60) and negatively associated with CD.

In addition, human interventional studies suggest a direct involvement of cocoa flavanols polymers in the colon microbiota modulation. In particular, higher growth of Bifidobacterium, Enterococcus and Lactobacillus species was shown to counteract *Clostridium histolyticum* gut colonization after a four-week cocoa beverage administration (494 mg polymeric proanthocyanidins/day) [107].

These complex interactions between microbial-derived cocoa compounds and gastrointestinal microflora are far from being completely understood because of the huge diversity of human colon microbiota and the limited possibilities of in vitro cultivation of all the human colon microorganisms. 

Taken together, these observations suggest that the beneficial effects of cocoa flavanols on human health discussed in this review are not only attributed to the main compounds but also to their metabolites. 

**Figure 3:**
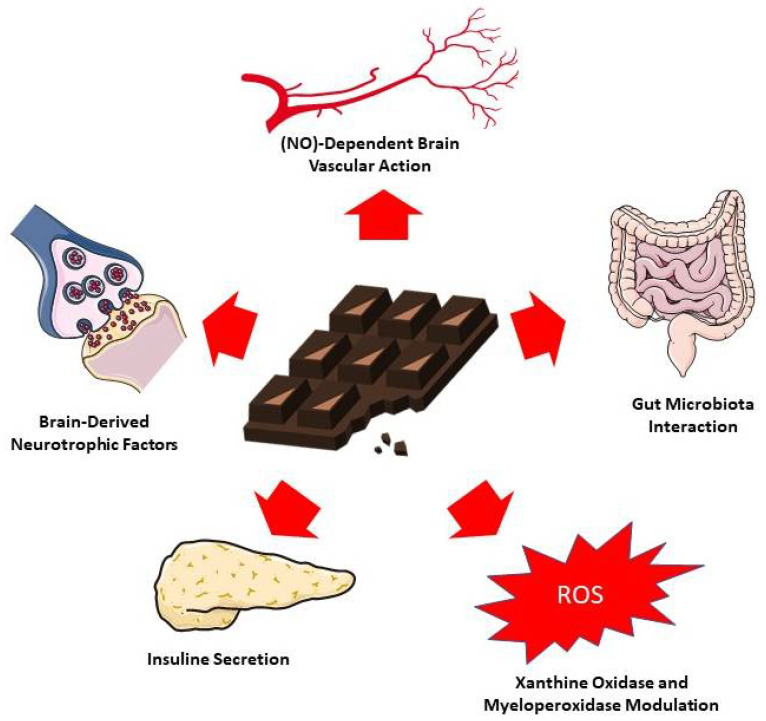
Proposed mechanisms for chocolate and cocoa action on cognition. The figure was partly generated using Servier Medical Art, provided by Servier, licensed under a Creative Commons Attribution 3.0 unreported license [108].

Figure 3 summarizes the main proposed mechanisms of action of cocoa-derived compounds on cognition.

## 5. Strengths and Limitations

We collected the results comprehensively and systematically; however, studies that did not include the chosen keywords in the title or abstract may not have emerged. However, they may regard secondary treatments or secondary endpoints. Moreover, we only used data from published results, though many ongoing trials may have generated unpublished data, as can be assumed from many entries without any results from ClinicalTrials.gov.

At present, we found heterogeneity in the results of the studies, and cognitive domains were measured in very heterogenic ways. The changes in general cognitive performances were often measured with MMSE, but this test is not sufficiently sensitive to reveal early signs of dementia [109]. MMSE should be better used as a measure of inclusion or exclusion criterion and administered only during the initial screening assessment. For future research, it could also be important to assess a more adequate set of tests in predicting conversion to CD.

Some trials had small sample sizes that do not permit extending their results to the general population and do not allow a sub-analysis (e.g., for gender).

Some observational studies have shown an effect that appears synergic between chocolate and other foods. In interventional studies, the baseline dietary flavanol intake may be a confounding factor that contributes to the magnitude of an individual’s response and can distort the interpretation of the results. Indeed, participants were not instructed to change or continue their usual lifestyle habits in all studies.

Moreover, the dietary patterns were assessed by self-administered food frequency questionnaires related to previous years, in which participants with impaired cognition may be less reliable. Furthermore, a clear quantification of food servings or a discernment among dark, milk or white chocolate were not always available.

Additionally, one must take into account the lack of a reliable dietary assessment (e.g., it was not known whether participants consumed a plant-based, Westernized or Mediterranean diet).

Another common limitation of observational studies using cocoa dairy-based drinks is the impact of milk on flavanols in chocolate and cocoa powder. Milk has been shown to reduce the absorption of antioxidants from chocolate [9].

Finally, some studies were sponsored by Mars Inc., a company with long-term research and commercial interests in chocolate products, which also supplied the standardized powdered cocoa drinks used in the investigations [53,54,55,56,57,60,66].

## 6. Conclusions

Observational studies are compatible with the role of cocoa and chocolate in cognition, though with some conflicting results. Chocolate consumption in epidemiological studies reflects very low flavan-3-ols doses against the high flavanols intake included in the RCTs. In this review, the main improvements in interventional studies in processing speed, executive function and working memory were observed when consuming cocoa drinks containing approximately 500–900 mg of flavanols. This evidence suggests a crucial role of the combination of time with concentration. Interestingly, the cognitive beneficial effects matched with improvements in blood pressure and insulin resistance. The relationship between health status and integrity of vascular and neurological functions is unknown. In light of the above considerations, cocoa and dark chocolate may be helpful for aged people to improve or recover their neurovascular connectivity. It seems that cocoa’s long-term cognitive protection could particularly affect populations at risk or with early CD compared to old people who are cognitively intact. The bioavailability of cocoa flavanols may differ in enriched products, such as cocoa drinks, compared to commercial chocolate. Dark chocolate has a significantly higher content of cocoa than milk chocolate, resulting in a higher intake of polyphenols. Future studies should pay close attention to chocolate types assessed in observational studies and used in interventions. In consideration of the fact that adequate safety with high acceptability emerged from the selected studies, the use of cocoa and chocolate appears promising for possible interventions in cognition in the elderly. Additional studies are needed to determine which ingredient, or combination, in chocolate and cocoa accounts for the effects observed. Cocoa, and especially chocolate, are considered energy-dense foods. However, their consumption appears to be easily managed in dietary interventions without affecting the weight gain. 

## Figures and Tables

**Table 1:** Main characteristics of selected observational studies.

Reference	Type of Study	n	Participants	Age	Duration	Outcomes	Main Findings
Nurk et al., 2009 [38]	Cross-sectional	2031	Healthy	70–74	/	Association with neuropsychological tests	Little cognitive improvement associated with chocolate
Crichton et al., 2016 [39]	Cross-sectional	968	Community-dwelling participants	23–98	/	Association with neuropsychological tests	Better scores among chocolate users without association with CD
Moreira et al., 2016 [40]	Prospective cohort	309	Healthy	>65	4 years mean of follow-up	MMSE score	Chocolate intake was associated with a lower risk of CD (RR = 0.59, 95% CI: 0.38–0.92)
Filippini et al., 2020 [41]	Case–control	54 + 54	EOD patients with their caregivers as control	65 as mean	/	Association between EOD and dietary factors	Lower risk of EOD with the upper level of chocolate use
Zhong et al., 2021 [42]	Prospective cohort	91,891	Patients with prostate, lung, colorectal and ovarian cancer	55–74	Up to 15.5 years of follow-up	Association between chocolate consumption and mortality	Strong inverse association between chocolate consumption and mortality for AD (HR = 0.69, 95% CI: 0.49–0.99)
Low et al., 2019 [43]	Nested Case–control	209 + 209	CD with matched controls	>65	Follow-up of 12 years	Metabolomics of diet-related biomolecules and cognition using MMSE and other neuropsychological tests	Cocoa metabolites were inversely associated with CD
González-Domínguez et al., 2021 [44]	Nested Case–control	209 + 209 and 212 + 212	CD with matched controls	>65	Follow-up of 12 years	Metabolomics of diet-related biomolecules and cognition using MMSE and other neuropsychological tests	Inverse association between 3-methylxanthine metabolite and subsequent CD
Haller et al., 2018 [49]	Prospective cohort	145	Healthy	69–86	Follow-up of 3 years	Neuropsychological tests and MRI	No correlation between chocolate consumption and cognition or MRI
Calabrò et al., 2019 [50]	Retrospective	55	MCI using Mexenion^®^	56–75	1 year	MMSE	Cocoa polyphenols intake was related to slowing down the cognitive worsening

**Table 2:** Main characteristics of selected interventional studies.

Reference	Type of Study	n	Participants	Age	Duration	Intervention	Outcomes	Main Findings
Sorond et al., 2008 [57]	Single-arm intervention	13	Healthy	59–83	2 weeks	900 mg cocoa flavanols drink per day	Transcranial Doppler ultrasound	Mean blood flow velocity increased after 1 or 2 weeks of flavanols intake
Sorond et al., 2008 [57]	Parallel RCT	21	Healthy	59–83	1 week	900 mg cocoa flavanols drink per day or placebo	Transcranial Doppler ultrasound	No cerebrovascular resistance or vasoreactivity improvements after the intervention. Mean blood flow velocity response increased in the intervention group but without statistical significance among groups
Sorond et al., 2013 [54]	Parallel RCT	60	With vascular risk factors, cognitively intact	>65	30 days	609 mg cocoa flavanols drink or 13 mg, two times per day	MMSE, cerebral blood flow velocity and MRI	Cocoa consumption was associated with neurovascular coupling and MMSE improvements in neurovascular coupling impaired patients
Desideri et al., 2012 [53]	Parallel RCT	90	MCI	71 ± 5	8 weeks	690, 520 or 45 mg cocoa flavanols drink per day	MMSE and TMT	Improvement in cognitive performance associated with cocoa use, also at an intermediate dosage
Mastroiacovo et al., 2015 [55]	Parallel RCT	90	Cognitively intact	61–85	8 weeks	690, 520 or 45 mg cocoa flavanols drink per day	MMSE, TMT and VFT	Improvement in cognitive performance associated with cocoa use, also at an intermediate dosage
Crews et al., 2008 [58]	Parallel RCT	101	Cognitively intact	>60	6 weeks	37 g dark chocolate with 397.30 mg proanthocyanins/g and 237 mL of cocoa beverage with 357.41 mg proanthocyanins/g per day or placebo (0.2 and 40.87 mg/g, respectively)	Neuropsychological test battery	No improvement after cocoa and chocolate intake
Suominen et al., 2020 [65]	Parallel RCT	100	Cognitively intact	65–75	8 weeks	50 g dark chocolate with 410 mg or 86 mg of flavanols per day	TMT and VFT	No improvement after cocoa and chocolate intake
Neshatdoust et al., 2016 [56]	Cross-over RCT	40	Healthy	62–75	12 weeks	494 mg or 23 mg flavanols cocoa drink per day	Serum BDNF levels, neuropsychological test battery	Significant increase in BDNF levels after high flavanols intake with improved cognitive function
García-Cordero et al., 2021 [59]	Parallel RTC	60	Healthy	50–75	12 weeks	Cocoa powder with 200 mg of flavanols per day, red berries mixture or both	Serum BDNF levels, neuropsychological test battery	No changes in BDNF levels. Neurocognitive enhancement in all groups
Brickman et al., 2014 [60]	Parallel RCT	41	Healthy	50–69	3 months	Cocoa with 900 mg or 45 mg of flavanol per day	CBV-fMRI, ModBent Task and mod Rey auditory learning task	Correlation between increased cerebral blood volume and better performance in the dentate gyrus after the high flavanol intake
Sloan et al., 2021 [66]	Parallel RCT	211	Healthy	50–75	12 weeks	260, 510 or 770 mg of cocoa flavanol capsules per day or placebo	CBV-fMRI, ModBent Task and mod Rey auditory learning task, List-Sorting Working Memory Test	Object-recognition list-sorting tasks were not improved after the intervention. An improvement in list-learning performance was associated with the cocoa intervention
